# Expectation of reward differentially modulates executive inhibition

**DOI:** 10.1186/s40359-019-0332-x

**Published:** 2019-08-23

**Authors:** Paula M. Herrera, Alberto Vélez Van Meerbeke, Mario Speranza, Claudia López Cabra, Mauricio Bonilla, Michaël Canu, Tristan A. Bekinschtein

**Affiliations:** 1 Grupo de Investigacion ‘Psiquiatria, Neurociencia y Comunidad’, Facultad de Medicina, Universidad Tecnológica de Pereira, Pereira, Colombia; 2 Grupo de investigación en neurociencias (NeURos), Universidad del Rosario, Bogotá, Colombia; 3 Consciousness and Cognition Lab, Department of Psychology, University of Cambridge, Cambridge, UK; 4 Laboratorio de Psicología Experimental, Facultad de Psicología, Universidad del Bosque, Bogotá, Colombia; 5 Departamento de Ingeniería Eléctrica, Universidad de Los Andes, Bogotá, Colombia; 6 HANDiReSP, Université de Versailles Saint Quentin en Yvelines, Le Chesnay, France

**Keywords:** Inhibition, Executive control, Expectation, Reward, Stop signal task

## Abstract

**Background:**

Inhibitory control, a key modulatory component of cognition guiding strategy and behaviour, can be affected by diverse contingencies. We explore here the effect of expectation of reward over behavioural adjustment in a Stop Signal Task modulated by reward. We hypothesize that cognitive control is modulated by different expectation of the reward.

**Methods:**

Participants were allocated to two groups differing in their degree of knowledge in what to expect from rewards. Expected Specific Reward participants (*N* = 21) were informed of the different monetary feedbacks they would receive after each successful inhibition. Unexpected Reward participants (*N* = 24) were only told that they would receive monetary reward after correct inhibitory trials, but not the amounts or differences.

**Results:**

Our results confirmed previous observations demonstrating a “kick-start effect” where a high reward feedback at the beginning of the task increases response inhibition. The Expected Specific Reward condition seems also to improve inhibitory control -as measured by the stop signal reaction time (SSRT)-, compared to the Unexpected Reward group.

**Conclusions:**

Knowledge of reward magnitudes seems to play a role in cognitive control irrespective of feedback magnitude. The manipulation of reward expectation appears to trigger different strategies for cognitive control, inducing a bottom-up effect of external cues, or a top-down effect given by the anticipation of incoming rewards. This is an early exploration to unearth possible higher order modulators - expectation and motivation- of cognitive control. This approach aims to gain insight into diverse psychopathological conditions related to impulsivity and altered reward systems such as Attention Deficit Hyperactive Disorder (ADHD), personality disorders, substance abuse, pathological gambling and cognitive aspects of Parkinson Disease.

**Electronic supplementary material:**

The online version of this article (10.1186/s40359-019-0332-x) contains supplementary material, which is available to authorized users.

## Background

The concept of inhibitory control in human cognition can be approached from its basic motor and reflexive aspects to elaborate control processes such as planned actions and strategies [1], it can also be simply defined as the resistance to interference [2]. From a cognitive perspective, inhibitory control is not only a fundamental tool to guide behaviour towards goals accomplishment but to dynamically modify or cancel planned actions [3]. This dynamic dimension of –inhibitory- cognitive control is crucial to enable the flexibility of cognitive and behavioural control systems [4].

**Fig. 1:**
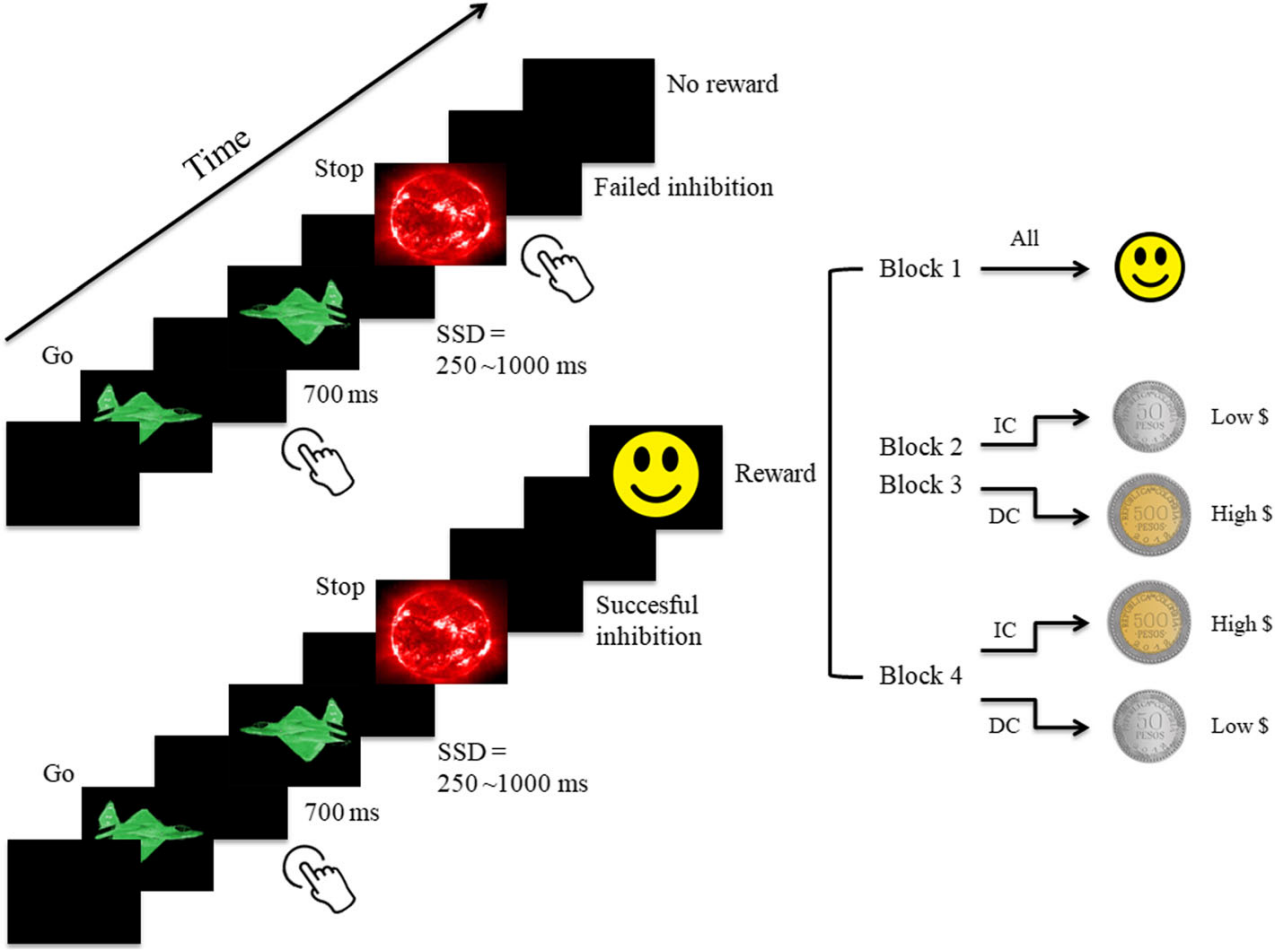
Experimental design. Reward Stop Signal Task (RSST). [Legend] Reward Stop Signal Task (RSST). All participants started with no monetary feedback and subsequently split in increasing (Smiley, 50$, 50$, 500$) or decreasing monetary (Smiley, 500$, 500$, 50$) reward conditions. *SSD* Stop signal delay, *IC* Increasing condition, *DC* Decreasing condition, Low $: low monetary reward (50$COP), High $: high monetary reward (500$COP).

The widely studied stop-signal task (SST) [5] has been instrumental in mapping characteristics of cognitive control in health and disease. In this task, when one of two signals appears on screen the appropriate response must be given, however, in a small proportion of trials a stop signal may appear (see Fig. 1); the aim is to avoid pressing the button, hence the name. The task is considered a *reactive control process* [1], but unlike the classical GoNoGo task [6] that depends on a direct reaction of immediate stimuli, the SST seems to recruit further central processes that require the withholding of the response as a strategy for performance. This process has been coined *proactive inhibition*, the ability to prepare to stop due to a possible upcoming signal. Internal states -goals and motivation- is thought to modulate proactive inhibition [7]. Moreover, some proposed that stopping implies a higher degree of complexity in the hierarchy of the control system, relying not only on motor inhibition but also on attention, memory and inner motivational states, the latest being closely related to the reward system [8]. Defining distinct control processes is still at the core of theoretical debates in cognitive neuroscience [3, 9], but despite this lack of consensus, it is widely accepted that the SST is a suitable and flexible tool for the exploration of cognitive control [10].

The extensive study of SST type of inhibitory control has led to partial understanding of the possible modulations that can be exerted over the system. The SST performance changes assume a top-down modulation by applying two rules, the go rule (performing an action), and the stop rule (refraining from pushing the button if you see the stop signal). Participants hold these two rules in mind to complete the task. A –further modulatory- third rule is introduced when participants are told that they can be rewarded or punished. This new rule may lead to a second-order level processing, changing the strategy of the participant by adding a motivational/expectation element to the application of the first two rules. Previous evidence supports this view [11–13] of a dynamic behavioural inhibition capacity in humans, as illustrated by the reward magnitude modulation and initial reward history effects [14].

Experiments applying subtle manipulations to the SST have been successful in showing distinct within-subject effects. In one hand, when similar stop signal patterns are presented, it is possible to induce a learning effect [15]; on the other hand, it has been described that the cost of inhibition failures induces a modulatory effect. If a punishment is introduced after a failed inhibition, the participant gets better Stop Signal Reaction Time (SSRT) scores and the number of failed stops drops [16]. This inhibitory improvement has also been reported when successful inhibition is rewarded [11–13]. There has been an increasing interest in the study of the role of reward over the modulation of inhibition, particularly in animal models of diseases [3], but in most human studies, participants have a single expectation of being rewarded with only one type of reward. Few experiments have directly explored the effect of distinct reward values in humans [17, 18], and even fewer have aimed to modulate expectations on the whole experiment [14].

In a previous study on a SST modified by reward levels, [16] we have suggested two effects of the monetary reward modulation over inhibition: an effect of the reward size itself -the kick start effect- induced by up-regulation of the highest reward at the beginning of the task. In this case, participants were aware of the reward magnitude assigned before performing the task, however, to test the second-order rule of expectation we decided to explore the strength of the influence induced by knowing the reward in advance, in contrast to only knowing the presence of reward.

In this work, we further present experimental evidence to confirm that the modulation of inhibition is affected by different reward magnitudes. However, beyond reward magnitudes itself, reward expectation seems to play a crucial role on the behavioural adjustment during an inhibition task [19, 20]. Other studies have proposed extra modulating parameters in a Horse Race model [21, 22] to explain finer dynamic adjustments to the Stop Task, when motivational aspects are manipulated. The so-called rational decision-making framework highlights the role of the sensory process and the action choice depending on the cost of inhibition errors [23], but it is hardly captured by the second-level processing proposed here.

The present work aims to contribute to the pending question about the role of expectation of reward on inhibition. We were interested in understanding the second-order level of the inhibitory control introduced with expectation: what happens when participants know they can win a high or a low reward but they are not aware of the order of the reward? And what happens if participants know about the presence of a monetary reward through the task but they are not told about the presence of different reward magnitudes?

One suitable hypothesis could be based on prediction error minimization [22]. In order to find the optimal strategy, the participant accumulates evidence that allows the identification of the minimal error probability and decision delay, leading to a decision threshold. Getting a reward during successful inhibitions could enhance the withholding strategies during a stop task. Then, it is tempting to push further the question about the reward magnitudes over the adjustment of behaviour.

In order to assess behavioural inhibition under expectation, participants performed our SST paradigm, modified by monetary reward levels, and distributed in two groups: the “expected specific rewards” group (ESRG), where participants were aware of distinct reward magnitudes, and the “unexpected reward” group (URG), where participants were only told about the presence of a monetary feedback. For the “expected specific rewards” group (ESRG), we hoped to replicate the kick-start effect when participants received a high reward at the beginning [14], and a modest effect of the reward magnitude itself. Whilst on the URG, we predict to find a kick-start effect over all participants at the first monetary reward block independently of the size of rewards, and an extra boost effect on performance over the blocks offering the highest reward.

## Methods

### Aim

In order to assess the effect of expectation over behavioural inhibition, participants performed a Reward Stop Signal Task (RSST), modified by monetary reward levels under expected (knowledge of specific reward magnitudes) and unexpected (only knowing there would be reward) reward conditions.

### Design

The general principle of Stop Tasks is a routine motor reaction where participants must hit a key each time they are confronted with a frequent go stimulus, and a cancellation of the ongoing action, after exposure to an infrequent stop signal. Our visual stimuli and experimental design consist on a modified version of the SST developed by Rubia and colleagues (2003) [24], which is, in turn, a faster visual variant of the Tracking SST [21]. Main modifications reside on the introduction of monetary feedback after each successful inhibition and the suppression of punishment feedback after a failed inhibition.

Participants performed the Reward Stop Signal Task Paradigm (RSST) in two different groups. One group was aware of the possibility of rewards magnitudes shift but the order of rewards was not communicated (ESRG). In the other group (URG), participants only knew that a monetary reward will appear without any mention to the reward shift and subsequently discovered -by themselves- a distinct reward magnitude only at the last block.

The RSST was presented over 4 blocks of 4 min each. Each block has one of the three possible feedbacks: non-monetary reward (Smiley), low reward (50$ COP –Colombian pesos-) or high reward (500$ COP). Regardless of the assigned condition or group, all participants performed exactly the same first – baseline- block, were each successful inhibition was rewarded with a Smiley. Afterwards, participants received two types of the mentioned monetary feedbacks.

To control for the effect of reward order presentation, we have built two conditions (See Fig. 1): for Increasing condition the order was Smiley, 50$ COP, 50$ COP, 500$ COP; and for Decreasing condition, Smiley, 500$ COP, 500$ COP, 50$ COP. Participants were randomly assigned to each condition in a counterbalanced way. Half of participants underwent the Increasing Condition and the other half, the Decreasing Condition.

The key point of the present experimental design was the difference in the information given about reward, ESRG expected different rewards magnitudes, and URG only knew that a reward will appear.

### Participants

Young adult participants were recruited by informal community announcements among undergraduate students attending at the Universidad El Bosque and at the Universidad del Rosario in Bogotá (Colombia). Forty-five participants were recruited from both universities ESRG group (*n* = 21) and the URG group (*n* = 24) resulted after randomisation. The combined mean age for both men and women participating from the study was 22.6. (Age range 20–31, sd =4.5). Sex ratio (w:m) in the “Expected rewards” group was 1.2, and in the “Unexpected rewards” group of 1.1 (more men).

Participants were screened for past and current psychiatric disorders. An open questionnaire was conducted in the search of history for Autism Spectrum Disorders, Learning Disorders, Attention Deficit Hyperactivity Disorder (ADHD), mood disorders (depression or bipolar disorders) and schizophrenia, as these were part of the exclusion criteria.

All time responses (mean reaction time [MRT], stop signal delay [SSD] and SSRT) were screened for outliers, given a cutting point of +/− 2 standard deviations from the mean response value (conservative threshold). Two participants were excluded from the study after applying these criteria.

### Materials

The task was programmed in Visual Basic 6.0 (pending link to the script here), a DELL personal computer with an Intell 2 processor was used to run the task. Participants were placed in a desk chair without wheels, at a standard distance of 1 m from a 20″ screen. Stimuli appeared against a black background at the centre of the screen. Alignment of the head was coordinated through visual verification, between the participant nose and the fixation cross at the centre of the screen. This alignment permitted to ensure that stimuli would be displayed in the middle of the visual field.

The testing room was artificially lighted, with no visual distractors on the walls and without windows, in order to avoid all attention-grabbers. At the beginning of the task, the participants underwent a short practice block, ensuring the correct visualization of every stimulus; luminosity was kept constant on the stimuli with no ambiguity.

### Procedure

Experimental environment conditions were controlled. All participants performed the experiment in a specific room arranged by each laboratory of experimental psychology at each University (del Rosario and El Bosque). Each room was equipped similarly, fulfilling the same conditions already described in *Materials*.

Instructions for the SST were presented in a standardized paper form and delivered by the same researcher. Participants were instructed that a video game-like task would determine how fast they were. They were told about the length of the task, comprising 4 blocks, with a short pause between blocks. After giving the instructions, participants were asked to repeat the procedure to the researcher in order to verify their full understanding of the task. When needed, questions were answered. A brief training block of the SST without feedback for successful inhibition was undertaken before beginning the trials.

### Data analyses

Statistical analyses were performed with R (R version 2.13.0 (2011-04-13) Copyright (c) 2011. The R Foundation for Statistical Computing). All data were checked for outliers, normal distribution and homogeneity of variance. Critical alpha was set at .05 (frequently adjusted using Bonferroni corrections) as guidance for interpretation of possible meaningful results.

As usual for the SST analysis, dependent variables consist on three type of response time measures (MRT, SSD, and SSRT), and four task performance measures (number of failed inhibitions, missed GO’s, wrong keys and number of rewards).

SSRT was generated through the mathematical model proposed by Logan and collaborators (Logan et al., 1997), following a subtraction of the MRT minus the SSD (formulae SSRT = MRT – SSD) [24], but see link to tools of the experiment.

We set to test the effect of magnitude of reward and order of reward. Also, key, the effect of expectation of rewards as the differences between knowledge of specific levels of reward (ESRG) vs. –simple knowledge of reward (URG) on inhibitory control.

To test the effect of order of rewards and the magnitude of rewards, we have conducted analyses through a two-way ANOVA given the within-subject factor ‘order of blocks’ (1,2,3… given by the acquisition block order), ‘type of reward’ (smiley, low reward, high reward) and between-subject factor ‘condition’ (Increasing reward, decreasing reward).

Then a General Linear Model (GLM) following a similar model as for the ANOVA, but including an extra level of analysis comparing groups: ESRG vs. URG. Post Hoc analyses were conducted through Bonferroni tests to compare differences between each block given their presentation timeline inside each condition (blocks (b) comparison as follows: b1-b2; b1-b3, b1-b4, b2-b3, b3–4). Alpha level set at 0.05.

These results allowed further testing of the effect of the dynamic progression of reward only in the SSRT, giving an insight over finer inhibition adjustments. To explore the degree of change between the first Block (Smiley, no monetary reward) and the subsequent monetary reward blocks, we conducted delta change comparisons (smiley/reward) through a Two-Way ANOVA based on the SSRT delta change ratio in percent between first block (Smiley/no monetary reward) and 2nd, 3th and 4ths blocks (with monetary reward). This analysis model allowed for a clear contrast on inhibitory performances between increasing vs. decreasing conditions among both groups (ESRG vs. URG) while parsing part of the variance contributed by the participants SSRTs to the initial –smiley- block.

These methods have been peer-reviewed prior to analyses (BMC Psychology).

## Results

### Expected specific reward group (ESRG)

#### Effect of order and reward magnitudes

Two-way ANOVA model was applied between ‘order of blocks’ inside each condition (increasing or decreasing reward). Main differences were observed between blocks comparisons on SSRT during decreasing condition, except between the 2nd and the 3th blocks. This is an expected outcome, given the fact that feedback was the same on blocks 2 and 3. No differences were found for SSRT for the increasing condition.

Two-way ANOVA conducted for reward magnitudes (smiley vs. low reward, smiley vs. high reward, low reward vs. high reward), revealed an effect of reward magnitude over SSRT between the non-monetary reward (smiley) and monetary rewards: between smiley vs. 1st high reward (F(1,19) = 2.6; *p* = .009), 2nd high reward (F(1,19) = 3.73; *p =* .004) and low reward on decreasing condition (F(1,19) = 3.2; *p =* .009).

**Fig. 2:**
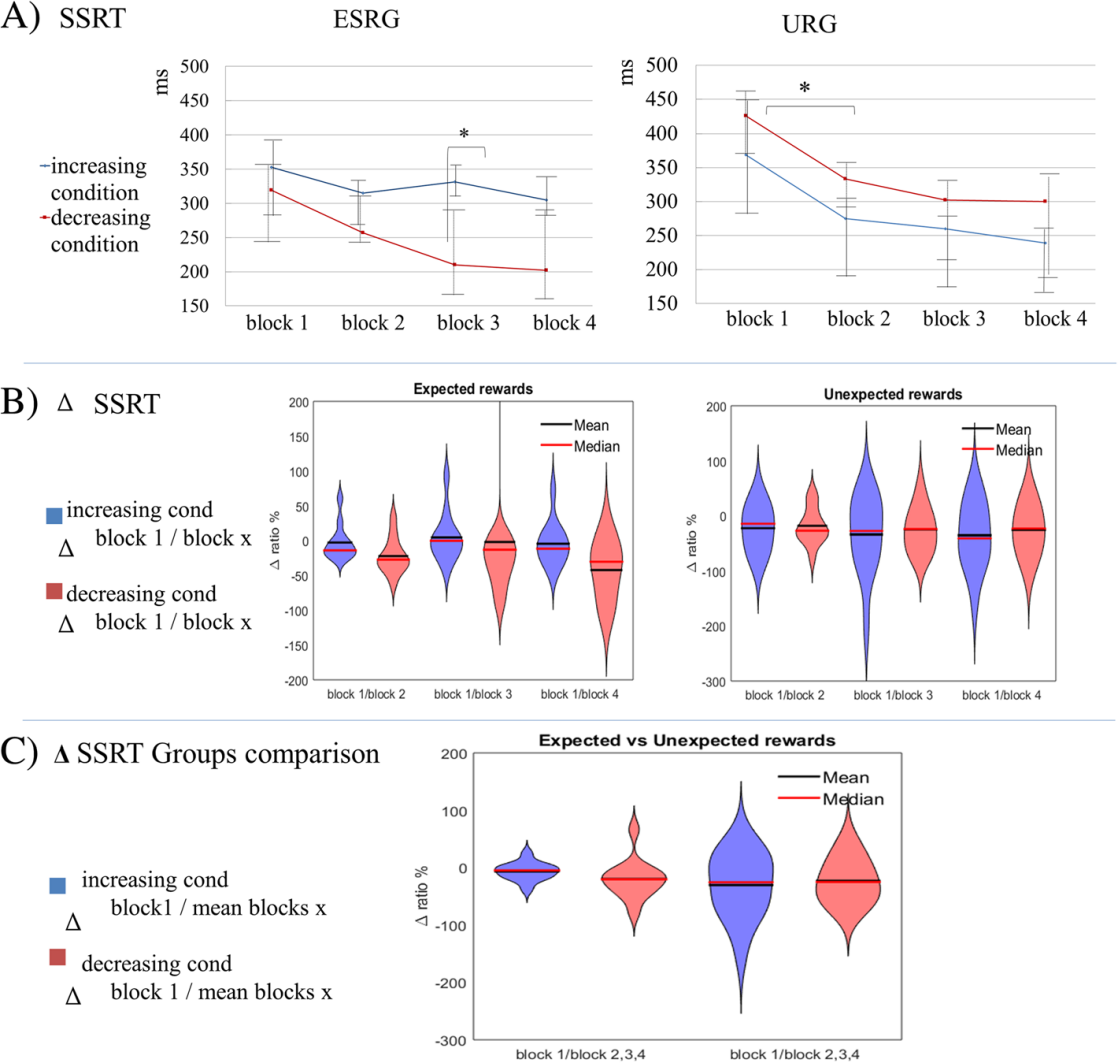
SSRT scores for Expected (ESRG) and Unexpected (URG) reward groups. [Legend] **a**. SSRT = stop signal reaction time, increasing condition on blue, decreasing condition on red. Left: (Expected reward shift group) Main differences were obtained on paired comparisons between the first no rewarded and the following rewarded blocks but only on the decreasing condition. Right (Unexpected reward shift group): main differences on both conditions were described between the first no rewarded and the following rewarded blocks.**b**. SSRT delta change ratio in percent between first block without monetary reward and 2nd, 3th and 4ths blocks with monetary reward. 2B Left: delta changes inside increasing and decreasing reward conditions on “Expected reward shift” group. * Significant difference between delta slopes comparing increasing and decreasing condition between 1st and 4th block (F(1,19) = 6.21; *p* = .022). 2B Right: delta changes inside increasing and decreasing reward conditions on “Unexpected of reward” group. No significant differences. **c**. SSRT delta change ratio in percent between Expected and Unexpected group, between Increasing and Decreasing reward conditions. *Difference was found between conditions on the “Expected reward” group on the delta change between the no monetary and the monetary rewarded blocks (F (1,19) = 5.74, (*p* = .027).

Two Way-mixed ANOVA was applied to explore the effect of the manipulation of reward orders given by the two reward order conditions (increasing Vs decreasing rewards), through the four blocks of the task (a 2*4 mixed model). Alpha level was set at .025. Main effect of group was found for SSRT (F(1,19) = 6.06, *p* = .001), but not reliable effect of Order or Group*Order interaction (See Fig. 2a for SSRT scores).

### Unexpected reward group (URG)

#### Effect of order and reward magnitudes

Two way ANOVA has revealed main differences over the transitions between the first and all the following blocks on SSRT values (F(1,22) = 8.9; *p* < .001). No differences were reported in paired comparisons including 2nd, 3th and 4th blocks.

Analyses for reward magnitudes inside each condition (increasing or decreasing) also highlighted a main effect on the transition between no monetary reward (smiley) and the first monetary reward. Moreover, inhibition scores given by the SSRT values reflect a robust effect of the presence of reward independently of being high or low and independent of its arrival order (SSRT between blocks (F(1,22) 1 and 2 = *p=,*004, block 1 and 3 = *p* = .005, block 1 and 4 = *p* = .003).

Two-Way mixed ANOVAs (α < .025) were performed to explore the effect of the manipulation of reward orders distributed over the two conditions (increasing vs. decreasing rewards), through the four blocks of the task (a 2*4 mixed model).

A robust effect of group was observed (SSRT = F(1,22) = 8.105; *p* < .001). There was no effect of order over performances, by the reward order of presentation. Furthermore, there was no effect of interaction between the variables and the order of presentation of rewards (“increasing or decreasing condition” independent factor). (For full results see Additional file 1 :Table S4).

### Non-monetary vs. monetary rewards

SSRT Delta ratio slopes were obtained through the comparison of percent change between the first block and each following block, computing the difference between Smiley condition (always first block) and the following 3 blocks (always monetary reward). Regarding ESRG, the Univariate GLM highlighted differences over SSRT delta scores between the 1st and the last block (block1 vs. block 4 = F (1,19) = 6.21, *p* = .022, effect size of 24% (Partial Etal Squared = .24). This steeper slope is clear on Fig. 2b for the “Expected reward group” (decreasing condition on red).

Regarding URG, no reliable difference was observed between the non-monetary and the monetary reward blocks, or between the increasing and decreasing conditions. The whole group had a similar progression pattern but no differences were retrieved among conditions (see Fig. 2b “Unexpected rewards” groups).

### Expected specific rewards vs unexpected rewards

Group analysis for blocks comparison between trials (Expected vs. Unexpected Reward shift) and between conditions (Increasing vs. Decreasing) through a Univariate GLM, was analysed, allowing the comparison of each time-corresponding block through both trials (blocks 1 on ESRG and URG groups, blocks 2 on both groups, and so on). No reliable effects were shown for SSRT measures.

A second Univariate GLM was conducted for trials and conditions comparisons, given each feedback value (smiley, low reward (50$ COP), high reward (500$ COP). A main effect of group was highlighted for each reward magnitude, under the ESRG vs. URG groups. No main effect of order of assignment of reward was seen, related to the increasing or decreasing reward condition.

Bonferroni post Hoc comparisons showed that main differences come from each first block and the following blocks, which highlights what was described earlier for each trial analysis: there is an important step on behaviour inhibition modulation between a non-monetary reward and the first monetary rewarded block. Moreover, delta changes analyses exhibited a main difference between the first block and the following ones, however only for the ESRG (F(1,19) = 5.74; *p =* 0.027. Effect size Partial Eta Squared = .232).

## Discussion

In the present study, we have conducted a stop signal task under two controlled conditions. First, we have introduced different reward magnitudes to manipulate the motivational dimension of the inhibitory task and evaluate the modulation of reward size. Second, we have tested the effect of the previous knowledge about reward over the behavioural adjustments in inhibitory control (ESRG vs. URG).

“Expected specific reward” experiment.

Results showed a main effect for each group on all variables, enabling us to confirm that the experimental manipulation has a modulatory effect over behaviour. Two main observations are made: there is an effect on inhibitory performances induced by the order of reward presentation, and an influence of the monetary reward magnitude.

The effect in the order of reward assignment was observed through differences between increasing and decreasing conditions. The increasing reward condition group exhibited a discrete change on the performance pattern, with a surprising less efficient inhibition score on the third block, and an expected -although modest- improvement at the end, when receiving the high reward.

Concerning the decreasing condition group, differences on pairwise tests on number of failed stops and SSRT scores were observed between the first block that gives non-monetary feedback (smiley) when compared to the subsequent monetary rewarded blocks. The dynamic progression of SSRT performance through the task exhibited a stronger delta change between the 1st and the 3th and 4th blocks on the decreasing condition group.

Given the presence of the high reward in the 2nd block, it was expected to also have a significant delta change at this point but this was not the case. Instead, it was observed a dynamic improvement of performance among the four blocks. Participants exhibited a better immediate global inhibition strategy under decreasing conditions in comparison with the increasing conditions. The presence of the strongest reward at the beginning of the monetary rewards seems to play a crucial role over inhibitory behaviour when participants are aware of the reward amounts. This initial boosting effect looks more substantial than the promise of a high reward later on the task. A similar kick-start effect has been described in a previous report on a Stop Signal Task [18], in which participants were told in advance about the exact moment when they were going to get a high or low reward. In contrast with the present work, inhibition performances mirrored the size of rewards: low reward, lower scores, higher reward, better performance.

By manipulating the knowledge of the reward type, we wanted to go beyond the kick-start effect and explore the modulation of expectations over executive inhibition. If we stick to the assumption that merely the reward size would be the cause of a performance boost, we could expect a simple replication of previous work. Although, current results in both conditions exhibit a non-linear progression that does not strictly follow the reward size modulation.

We can claim that expectations about rewards to come modify the way participants adjust their inhibitory strategies.

Given the manipulation of information about reward shift, we have certainly induced an expectation that works through the ongoing task course. When participants “discover” the size of the reward at the second block, they can predict what is coming next, would it be another high reward or a low one. At the third block, the prediction becomes a certainty: if you get another high reward at that point, there is no doubt that the reward size shift will come in the fourth block.

Regarding the increasing conditions group, we may have induced an undermining effect. The presence of a low reward, when you are expecting a high one, may have acted as a demotivating or non-attractive reward. This demotivation is perceived through a lack of improvement when compared to the group receiving the high reward in same moment of the task. The undermining effect seems to be confirmed by the third block, when participants are confronted again with a low reward, showing worse inhibition scores than the previous block. Finally, when these participants received the highest reward inhibition scores improve, with a reliable difference between the first and the last block on number of obtained rewards.

“Unexpected reward” experiment.

Analyses showed a main effect of group on all time performance variables (MRT, SSD, SSRT), and over failed stops, number of rewards and number of wrong keys, which confirms the modulation effect on behaviour induced by the experimental manipulation.

Following the same procedure as for the “Expected specific reward” group, participants did not realize that they belonged to one of the two existent conditions (increasing and decreasing reward). Additionally, we have suppressed the information about the existence of distinct reward magnitudes in this group.

As expected, with reward, results showed a progressive improvement on all measured scores, independently of the condition, and regardless to the reward magnitude order. This supports a general kick-start effect on performance, after the introduction of a monetary reward on the task. The presentation of the $500 coin may have induced a stronger stability on the prediction system, reflected on significant shorter SSD on MRT scores after the first blocks.

Slopes between blocks and between the two conditions were quite similar for all measures. The steeper slope was observed between the first and the second block. This corresponds to the shift between a non-monetary feedback (smiley) and the first monetary reward, no matter if it concerned a low or a high reward. These findings confirm the hypothesis that on unexpected reward shift trials, the modulation effect of monetary reward would be induced by the presence of the reward itself, independently of its magnitude. Moreover, the lack of information about rewards shift restrains a possible dynamic modulation of expectation through the task. The hypothesis about the reward magnitude effect per se was not confirmed. An extra boost of high reward at the end, or an undermining effect of the low reward was not observed. Instead, we can claim the up modulation of the inhibitory system by the mere presence of any amount of monetary reward. Instructions given to the “unexpected reward” group may have induced a single boost in expectation that worked as inner motivation, placing any amount of money at the top of expected feedbacks (supported by informal conversation with participants). The kick-start effect may have operated in a similar way as the one induced by the presence of explicit high reward at the beginning of the task.

The expected reward shift effect can be partially assimilated to the effect of the anticipation of reward. Previous studies have stated that the announcement of high rewards for future performances inside a consecutive set of tasks can boost performance during intermediate tasks involving interference control (The Simon task [25]). The anticipation of reward boosting mechanism could be perceived in distinct behavioural adjustments depending on the type of experiment even if they belong to the generic group of inhibition paradigms [26–28]. Our results do not replicate this observation directly as we are using another task, but they also do not support a direct converge in the results. Instead, we have observed an immediate and steady effect of the highest reward at the beginning of the task, when previous information or previous assumptions are made about the size of expected rewards. Participants receiving low rewards at the beginning did not exhibit any immediate performance boost on low reward blocks when higher later rewards were expected. These results seem more in tune to the undermining effect [29]. Receiving a low reward when you expect a high one at some point may have induced a demotivation, similar to the removal of an attractive reward. In the present work we have used the stop signal task with a dynamic algorithm adjusting to each subject performance in order to avoid learning strategies. This another reason to keep the term “kick-start effect” as a distinct concept than anticipation of rewards, because no extra boost performance was observed in intermediate blocks while waiting for the biggest bonus. We propose that these two concepts correspond to distinct neural mechanisms. The boosting effect of a strong reward at the beginning of a task has not been explored in motor/cognitive inhibition tasks like ours.

The “kick-start effect” that we describe here could induce a more immediate up regulation of dopamine release in a phasic pattern, stirred by a quick and salient monetary cue [30]. However, we speculate that the anticipation of rewards would rely on more complex cognitive circuits demanding prefrontal involvement through goal-directed behaviour [31] possibly via subcortical- ventral striatum circuits [32] mediated in part by a tonic dopamine release [33].

Apparently, when participants are told in advance about the different reward sizes, this information may induce a stronger influence of the order of presentation of rewards. Hence, we can also claim that “expectation matters”. In everyday life, this can be reflected on video games personal choice. Even if you don’t receive real money or gifts, people look for the excitement of winning any kind of reward: coins, candies, some aliens or zombies to kill [34, 35]. What seems to matters here is the inner value you give to what you expect to get, according to intrinsic motivation.

Furthermore, our results are consistent with –for example- predictive coding theory [36–38]. Analyses showed steadier values on the first blocks, accounting for only limited information of the task on both trials. After the first block, we assume that a screening system of outcomes is in place, waiting for the highest reward appearance. Moreover, on the unexpected reward shift group, a single reward is expected without any other specific belief, participants are not aware of the presence of reward magnitude differences. Variances are also smaller in the first block, and beyond that, they become high and unstable, except on decreasing conditions, when the high reward at the beginning can be perceived as “strong enough” (500$ enticing Colombian pesos), which in turn may be interpreted as higher precision induced by the reward. All these observations are also consistent –alternatively- with the hypothesis proposed by Ide and colleagues about rational decision-making in inhibitory control [4], based on optimal prediction of outcomes that modulate inhibitory behaviour.

## Conclusions

Three main components of inhibition can be tested with the Stop Signal Task modified by reward levels: the motor/proactive inhibition, the cognitive inhibition and the influence of motivation. The cognitive aspect is given by an overt instruction to restrain the action under a very specific circumstance, leading to several requirements, first the understanding of the rule given by verbal instruction, and then the requisite of retention of information in working and short-term memory. The following withholding strategy seems enhanced by motivation, in addition to the pro-active/planned inhibition capacity [8]. The motivational aspect is driven by modulations of performances in front of distinct types of rewards, monetary or not.

Here we claim that expectation may constitute an extra element to consider when testing the motivational effect of reward over cognitive control, inducing an influence on both the cognitive dimension as well as on the inner motivation.

In the present work, we have addressed the question about the effect of expectation on a rewarded inhibitory task by asking if there is a difference on performance when participants know they can win a high or a low reward, compared to participants that do not know about reward differences. The answer is yes. On one side, a robust effect was obtained through analyses comparing increasing and decreasing conditions inside the “Expected specific reward” group. Participants receiving the highest reward at the beginning of the task have modulated the inhibition pattern in a more efficient way than those starting with low reward. Moreover, the dynamic progression of SSRT scores on the decreasing condition was similar to a previous study reporting the kick-start effect. Even if the order of the reward shift was unknown, participants received the higher reward at the beginning and were able to deduce what amount of money could come next. On the other hand, participants not knowing about the presence of distinct reward magnitudes reached a ceiling pattern right after receiving the first reward, would it be high or low. This observation suggests that participants only had a single high-level prediction: receiving monetary reward. Moreover, the behavioural adjustments for unexpected reward shift were similar to the one exhibited by the participants only assessed in the kick-start effect in the previous study. Allegedly, the highest performance effort was allocated promptly at the bonus arrival moment.

“As long as you get paid” seems good enough for modulating inhibition, as stated by some studies [11, 15, 17]. The novelty of the present work is given by the observation that expectations about rewards seem to induce a stronger effect than the reward magnitude itself, or if theorised that it is processed at a higher prediction level, that it drives the response.

Uncertainty is an influential factor over behavioural modulations, although it is difficult to build up controlled experimental conditions to test it [39]. Some authors have proposed computational models to assess adaptive behaviour [38].

The experimental design proposed here could be employed as an evaluation tool to assess behavioural adjustments for rewards in an uncertain environment. Although our results are preliminary, they may serve as an initial guide to understand the effect of expectation over inhibitory processes.

## Additional file

Additional file 1: **Table S1.** Behavioural data on all variables for “Expected specific rewards” group (ESRG). M (mean) and SD (standard deviation). Reaction times are represented of MRT (mean reaction time), SSD (Stop signal delay), and SSRT (Stop Signal Reaction Time). **Table S2.** Reward Stop Signal Task (RSST). Expected Specific Reward group (ESRG). Two-Way mixed ANOVA for time and task performance measures. **Table S3.** Behavioural data on all variables for “Unexpected reward” group (URG). M (mean) and SD (standard deviation). Reaction times are represented of MRT (mean reaction time), SSD (Stop signal delay), and SSRT (Stop Signal Reaction Time). **Table S4.** Reward Stop Signal Task (RSST). Unexpected group (URG). Two-Way ANOVA for time and task performance measures. **Table S5.** Reward Stop Signal Task (RSST). Expected and Unexpected [ESRG - URG] group analyzes. Between conditions [Increasing Vs Decreasing]. GLM Univariate between blocks [effect of order] Group analysis for block comparison between groups [ESRG Vs URG]. **Table S6.** Reward Stop Signal Task (RSST). Group analysis for block comparison between groups [ESRG Vs URG] and between conditions [Increasing Vs Decreasing]. GLM Univariate between blocks [effect of order]. **Table S7.** Group analysis for reward magnitude comparison between groups [ESRG Vs URG] and between conditions [Increasing Vs Decreasing]. GLM Univariate between rewards [effect of reward magnitudes] **Figure S1.** Time performances for Expected (ESRG) and Unexpected (URG) reward groups. (PDF 445 kb)

## Data Availability

Data is available at the University of Cambridge Repository (10.17863/CAM.6920).

## Abbreviations

ADHD: Attention deficit hyperactive disorder
ESRG: expected specific rewards group
MRT: mean reaction time
RSST: reward stop signal task
SSD: stop signal delay
SSRT: stop signal reaction time
SST: stop signal task
URG: unexpected rewards group
