# Supplementary material for: Expectation of reward differentially modulates executive inhibition
Source: BMC Psychol. 2019 Aug 23;7:55. doi: 10.1186/s40359-019-0332-x (PMC6706938; doi:10.1186/s40359-019-0332-x)
Supplement: Supplementary file 1 — Table S1. Behavioural data on all variables for “Expected specific rewards” group (ESRG). M (mean) and SD (standard deviation). Reaction times are represented of MRT (mean reaction time), SSD (Stop signal delay), and SSRT (Stop Signal Reaction Time). Table S2. Reward Stop Signal Task (RSST). Expected Specific Reward group (ESRG). Two-Way mixed ANOVA for time and task performance measures. Table S3. Behavioural data on all variables for “Unexpected reward” group (URG). M (mean) and SD (standard deviation). Reaction times are represented of MRT (mean reaction time), SSD (Stop signal delay), and SSRT (Stop Signal Reaction Time). Table S4. Reward Stop Signal Task (RSST). Unexpected group (URG). Two-Way ANOVA for time and task performance measures. Table S5. Reward Stop Signal Task (RSST). Expected and Unexpected [ESRG - URG] group analyzes. Between conditions [Increasing Vs Decreasing]. GLM Univariate between blocks [effect of order] Group analysis for block comparison between groups [ESRG Vs URG]. Table S6. Reward Stop Signal Task (RSST). Group analysis for block comparison between groups [ESRG Vs URG] and between conditions [Increasing Vs Decreasing]. GLM Univariate between blocks [effect of order]. Table S7. Group analysis for reward magnitude comparison between groups [ESRG Vs URG] and between conditions [Increasing Vs Decreasing]. GLM Univariate between rewards [effect of reward magnitudes] Figure S1. Time performances for Expected (ESRG) and Unexpected (URG) reward groups. (PDF 445 kb) [file 40359_2019_332_MOESM1_ESM.pdf]

Expectation of reward differentially modulates executive inhibition

Paula M. Herrera[1,2,3,6], Alberto Vélez Van Meerbeke[2], Mario Speranza[1], Claudia López Cabra [2], Mauricio Bonilla [4], Michaël Canu[5] and Tristán A. Bekinschtein[3]

[1] Universidad Tecnológica de Pereira. Facultad de Medicina, Departamento de Psiquiatría

[2]Grupo de investigación en neurociencias (NeURos), Universidad del Rosario, Bogotá, Colombia

[3] Consciousness and Cognition Lab, Department of Psychology, University of Cambridge, United Kingdom.

[4] Laboratorio de Psicología Experimental, Facultad de Psicología, Universidad del Bosque, Bogotá, Colombia

[5] Departamento de Ingeniería Eléctrica. Universidad de Los Andes, Bogotá, Colombia

[6] HANDiReSP, Université de Versailles Saint Quentin en Yvelines, Le Chesnay, France

Corresponding author:

Paula M. Herrera[1,2,3,6]

Email Address: p.herrera@utp.edu.co

Additional results

Additional Table 1. Behavioural data on all variables for "Expected specific rewards" group (ESRG). M (mean) and SD (standard deviation). Reaction times are represented of MRT (mean reaction time), SSD (Stop signal delay), and SSRT (Stop Signal Reaction Time)

Supplementary Table 1. Stop Signal Task on Expected reward group. Increasing and decreasing conditions

Order	Increasing Condition (n=11)				Decreasing Condition (n=10)			
	1	2	3	4	1	2	3	4
Reward Size	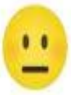	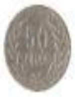	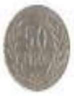	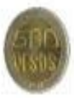	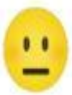	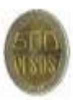	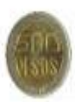	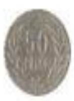
	M/*SD*	*M*/*SD*	*M*/*SD*	*M*/*SD*	*M*/*SD*	*M*/*SD*	*M*/*SD*	*M*/*SD*
MRT (ms)	723.17	841.81	872.54	896.91	849.63	905.99	891.62	878.09
	205.33	256.41	269.85	241.23	225.45	220.23	207.63	216.14
SSD (ms)	370.53	526.71	541.12	592.07	530.63	649.1	681.86	676.22
	291.58	298.34	301.49	254.99	225.31	191.64	216.34	250.28
SSRT (ms)	352.64	315.1	331.42	304.84	318.99	256.89	209.77	201.87
	133.61	98.65	97.93	97.46	147.75	126.42	185.35	191.64
Failed Stops	13.1	12.4	11.9	10.7	12.09	10	8.55	9.27
(n°)	6.71	6.54	4.8	4.27	3.27	3.13	4.74	4.25
Missed Go's	3.3	3.4	3	2.5	4.82	4.64	4.18	4.27
(n°)	3.92	3.86	2.11	2.01	3.68	3.7	3.84	2.41
Wrong Keys	3.2	1.5	1.1	1	5.36	5.09	5.82	5.91
(n°)	2.39	1.18	1.29	0.67	11.92	11.66	11.51	11.72
Rewards	14.8	15.6	16.7	18.6	13.55	15.36	15.09	15.36
(n°)	6.53	6.55	5.03	4.12	3.88	5.61	6.44	4.78

All measures of performance in speed an accuracy.
MRT, mean reaction time; SSD, Stop signal delay; SSRT, Stop signal reaction time.

Additional Table 2. Reward Stop Signal Task (RSST). Expected Specific Reward group (ESRG). Two-Way mixed ANOVA for time and task performance measures

	**Main effect of group**	**Main effect of order**	**Group*Order interaction**
	(df=3,57) *F(p)*	*(df=1,19)* *F(p)*	*(df=3,57)* *F(p)*
**MRT (ms)**	***5,61 (0,003) ****	0,26 (0,62)	2,21 (0,096)
**SSD (ms)**	***13,10 (<0,001) *****	1,48 (0,23)	0,50 (0,68)
**SSRT (ms)**	***5,72 (0,002) *****	1,99 (0,17)	1,85 (0,14)
**Failed stops**	***3,87 (0,014) ****	***6,93 (0,016) ****	.75 (,523)
**Missed go's**	0,475 (0,701)	1,15 (0,29)	0,082 (0,97)
**Wrong keys**	***0.28 (0,043) ****	2,79 (0,11)	***5,76 (0,002) ****
**Rewards**	***3,23 (0,029) ****	***7,78 (0,012) ****	0,93 (0,43)

MRT, mean reaction time; SSD, stop signal delay; SSRT, stop signal reaction time. Order refers to the reward order assignment for each condition group.
Failed stops, missed go's, wrong keys, rewards on absolute number of events.
Bonferroni posthoc comparison for Failed stops: significant for block 1 – block 4 (p=.04), for Wrong keys: trend between block 1-block 2(p=.072), for Rewards, difference between block 1 and block 4(p=.024).

Additional Figure 1. Time performances for Expected (ESRG) and Unexpected (URG) reward groups.

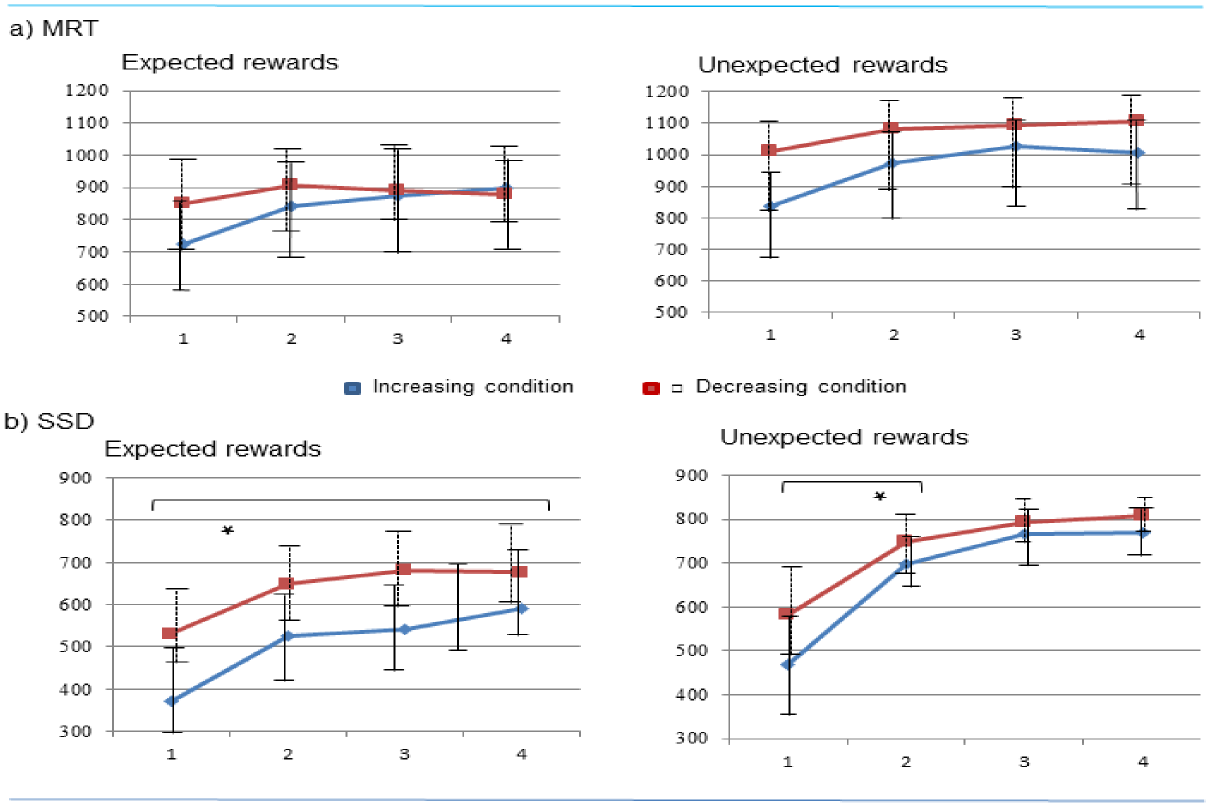

MRT=mean reaction time, SSD= Stop signal delay, SSRT= stop signal reaction time, increasing condition in blue, decreasing condition in red. Main differences on SSD and SSRT scores between blocks on ESRG group. Main differences between no monetary and first monetary reward (Smiley Vs coin) on SSD and SSRT on URG group.

Additional Table 3. Behavioural data on all variables for "Unexpected reward" group (URG). M (mean) and SD (standard deviation). Reaction times are represented of MRT (mean reaction time), SSD (Stop signal delay), and SSRT (Stop Signal Reaction Time)

Supplementary Table 2. Stop Signal Task on Unexpected reward group. Increasing and decreasing

Order	Increasing Condition (n=11)				Decreasing Condition (n=12)			
	1	2	3	4	1	2	3	4
Reward Size	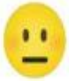	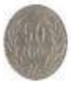	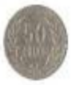	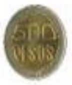	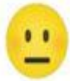	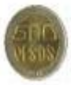	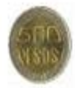	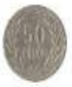
	M/*SD*	M/*SD*	M/*SD*	M/*SD*	M/*SD*	M/*SD*	M/*SD*	M/*SD*
MRT (ms)	837.2	972.8	1027.21	1007.89	1008.83	1081.2	1093.92	1108.14
	223.83	219.4	232.96	229.64	178.42	176.35	172.94	176.17
SSD (ms)	468.42	697.64	767.35	769.02	582.73	747.89	791.91	808.2
	287.77	115.07	108.05	103.81	258.83	134.17	76.51	43.4
SSRT (ms)	368.78	275.16	259.86	238.87	426.09	333.31	302.01	299.94
	141.77	127.99	188.35	165.58	111	106.16	136.29	160.88
Failed Stops	14.67	11.25	9.08	8.5	14	11.42	10.33	8.17
(n°)	4.58	2.93	3.99	3.87	5.59	2.81	3.6	3.64
Missed Go's	6	3.25	3.08	2.58	5.33	5.92	4.67	3.67
(n°)	5.88	2.83	2.15	1.62	6.51	7.85	3.73	2.02
Wrong Keys	2.42	1	1.92	1.5	1.92	1.42	1.42	0.75
(n°)	2.28	1.28	1.78	2.2	1.44	1.73	1.93	0.75
Rewards	14.33	18.17	21	22	15.33	17.75	19.58	21.83
(n°)	4.83	3.19	3.89	3.81	5.25	4.85	3.92	3.69

All measures of performance in speed an

MRT, mean reaction time; SSD, Stop signal delay; SSRT, Stop signal

Additional Table 4. Reward Stop Signal Task (RSST). Unexpected group (URG). Two-Way ANOVA for time and task performance measures

	Main effect of group	Main effect of order	Group*Order interaction
	(df=1,22)	*(df=1,22)*	*(df=3,66)*
	F(p)	*F(p)*	*F(p)*
**MRT (ms)**	***2,57 (0,061)***	***12,72 (0,002)****	1,48 (0,22)
**SSD (ms)**	***2,93 (0,040)****	2,18 (0,15)	1,00 (0,39)
**SSRT (ms)**	***8,90(<0,001)*****	0,103 (0,75)	1,42 (0,24)

MRT, mean reaction time; SSD, Stop signal delay; SSRT, Stop signal reaction time. Order refers to the reward order assignment for each condition group

Additional Table 5. Reward Stop Signal Task (RSST). Expected and Unexpected [ESRG - URG] group analyzes. Between conditions [Increasing Vs Decreasing]. GLM Univariate between blocks [effect of order] Group analysis for block comparison between groups [ESRG Vs URG]

	Blocks	Main effect of Group [ESRG Vs URG]	Main effect of order [IC vs DC]	Group[Expected Vs Unexpected]* Order [IC vs DC]
		(df=1,41)	*(df=1,41)*	*(df=3,44)*
		F(p)	*F(p)*	*F(p)*
**MRT (ms)**	*Block 1*	***4,78 (0,034)****	***5,36(0,02)****	0,13(0,71)
	Block 2	***5,52 (0,024)****	1,75(0,19)	0,15(0,73)
	Block 3	***7,26 (0,01)****	0,42(0,52)	0,12(0,72)
	Block 4	***6,97 (0,01)****	0,39(0,53)	0,85(0,36)
**SSD(ms)**	*Block 1*	0,88 (0,35)	2,95(0,09)	0,08(0,77)
	Block 2	***5,51 (0,02)****	2,26(0,14)	0,39(0,53)
	Block 3	***8,76 (0,005)*****	2,11(0,15)	1,04(0,31)
	Block 4	***8,10 (0,007)*****	1,29(0,26)	0,17(0,68)
**SSRT (ms)**	*Block 1*	2,36 (0,13)	0,08(0,76)	1,29(0,26)
	Block 2	0,27 (0,60)	0,00(0,99)	2,81(0,10)
	Block 3	0,048 (0,82)	0,70(0,40)	3,00(0,09)
	Block 4	0,11 (0,73)	0,19(0,66)	2,97(0,09)

MRT, mean reaction time; SSD, Stop signal delay; SSRT, Stop signal reaction time. Order refers to the reward order assignment for each condition group

Additional Table 6. Reward Stop Signal Task (RSST). Group analysis for block comparison between groups [ESRG Vs URG] and between conditions [Increasing Vs Decreasing]. GLM Univariate between blocks [effect of order]

	Blocks	Main effect of Group [ESRG Vs URG]	Main effect of order [IC vs DC]	Group [ESRG Vs URG]* Order [IC vs DC]
		(df=1,41)	*(df=1,41)*	*(df=3,44)*
		F(p)	*F(p)*	*F(p)*
**MRT (ms)**	*Block 1*	***4,78 (0,034)****	***5,36 (0,02)****	0,13 (0,71)
	Block 2	***5,52 (0,024)****	1,75 (0,19)	0,15 (0,73)
	Block 3	***7,26 (0,01)****	0,42 (0,52)	0,12 (0,72)
	Block 4	***6,97 (0,01)****	0,39 (0,53)	0,85 (0,36)
**SSD(ms)**	*Block 1*	0,88 (0,35)	2,95 (0,09)	0,08 (0,77)
	Block 2	***5,51 (0,02)****	2,26 (0,14)	0,39 (0,53)
	Block 3	***8,76 (0,005)*****	2,11 (0,15)	1,04 (0,31)
	Block 4	***8,10 (0,007)*****	1,29 (0,26)	0,17 (0,68)
**SSRT (ms)**	*Block 1*	2,36 (0,13)	0,08 (0,76)	1,29 (0,26)
	Block 2	0,27 (0,60)	0,00 (0,99)	2,81 (0,10)
	Block 3	0,048 (0,82)	0,70 (0,40)	3,00 (0,09)
	Block 4	0,11 (0,73)	0,19 (0,66)	2,97 (0,09)

MRT, mean reaction time; SSD, Stop signal delay; SSRT, Stop signal reaction time. Order refers to the reward order assignment for each condition group

Additional Table 7. Group analysis for reward magnitude comparison between groups [ESRG Vs URG] and between conditions [Increasing Vs Decreasing]. GLM Univariate between rewards [effect of reward magnitudes]

	Rewards	Main effect of Group [ESRG Vs URG]	Main effect of order [IC vs DC]	Interaction between Order [IC vs DC] Group [ESRG Vs URG]
		(df=3,57)	*(df=1,19)*	*(df=1,19)*
		F(p)	*F(p)*	*F(p)*
**MRT (ms)**	***Smiley***	***4,78 (0,034)****	***5,36 (0,02)****	0,13 (0,71)
	50$	***7,74 (0,008)****	1,75 (0,19)	0,58 (0,45)
	500$	***4,86 (0,033)****	0,40 (0,53)	0,24 (0,62)
**SSD(ms)**	***Smiley***	0,88 (0,35)	2,95 (0,09)	0,08 (0,77)
	50$	***6,60 (0,014)****	***4,86 (0,033)****	0,10 (0,74)
	500$	***6,87 (0,012)****	0,11 (0,73)	0,55 (0,46)
**SSRT (ms)**	***Smiley***	2,36 (0,13)	0,08 (0,76)	1,29 (0,26)
	50$	0,42 (0,52)	0,97 (0,33)	2,37 (0,13)
	500$	0,019 (0,89)	0,36 (0,54)	3,46 (0,07)

MRT, mean reaction time; SSD, Stop signal delay; SSRT, Stop signal reaction time. Order refers to the reward order assignment for each condition group

Additional discussion

As already said about the structure of the SST, two main components of inhibition can be tested with this task: the motor/reactive and the cognitive inhibition. The cognitive aspect is given by an overt instruction to restrain the action under a very specific circumstance, leading to several requirements: first the understanding of the rule given by verbal instruction and then requiring the retention of information on working memory. The following withholding strategy seems enhanced by motivation, besides to the pro-active/planned inhibition capacity, relied on specific neural structures. Once the Stopping signal is detected and decoded, the right Inferior Frontal cortex and the Dorso Medial Frontal Cortex (preAdditional motor area – preSMA) trigger the inhibitory signal that goes to the Basal Ganglia and from there, inhibits the PreMotor area (M1) [1]. Implication of dorsolateral prefrontal cortex (DLPFC) during the stop task has been correlated with the working memory requirements to remember an instruction on experimental conditions. So, it has been stated that it is possible to anticipate the stopping strategies [2;3].

Beyond the influence of the reward itself over behaviour modulation, our results also suggest other subtle effects given by the knowledge of the reward size, and the expectation of the reward outcome. Recent studies support this hypothesis through neuroimaging evidence [4]. We can claim that there is a remarkable effect of the highest reward presented at the beginning of the task, on subjects that know the different reward sizes. This phenomenon, that we have called the “Kick start effect”, seems to be provoked by two possible pathways: by expectation (when subjects are told about the reward magnitudes) and by visual perception of the reward, after a no rewarded experience. A

deeper exploration of physiological variables could help to explore more subtle, subliminal effects of the reward magnitudes, as already suggested by Pessiglione and collaborators [5].

Additional references

1. Aron AR, Fletcher PC, Bullmore ET, Sahakian BJ, & Robbins, TW. Stop-signal inhibition disrupted by damage to right inferior frontal gyrus in humans. Nature neuroscience. 2003;6:2.

2. Jahfari S, Stinear CM, Claffey M, Verbruggen F, & Aron AR. Responding with restraint: what are the neurocognitive mechanisms? Journal of cognitive neuroscience.2010;22:7.

3. Chikazoe J, Jimura K, Hirose S, Yamashita KI, Miyashita Y, & Konishi S. Preparation to inhibit a response complements response inhibition during performance of a stop-signal task. The Journal of Neuroscience. 2009;29:50.

4. Lebreton M, Bertoux M, Boutet C, Lehericy S, Dubois B, Fossati P, & Pessiglione M. A critical role for the hippocampus in the valuation of imagined outcomes. PLoS Biol. 2013;11:10.

5. Pessiglione M, Schmidt L, Draganski B, Kalisch R, Lau H, Dolan RJ, & Frith CD. How the brain translates money into force: a neuroimaging study of subliminal motivation. Science. 2007;316:5826.
